# Spatio-temporal genetic variation of the biting midge vector species *Culicoides imicola* (Ceratopogonidae) Kieffer in France

**DOI:** 10.1186/s13071-016-1426-4

**Published:** 2016-03-11

**Authors:** Stéphanie Jacquet, Karine Huber, Hélène Guis, Marie-Laure Setier-Rio, Maria Goffredo, Xavier Allène, Ignace Rakotoarivony, Christine Chevillon, Jérémy Bouyer, Thierry Baldet, Thomas Balenghien, Claire Garros

**Affiliations:** Cirad, UMR15 Contrôle des Maladies Animales Exotiques et Emergentes, Campus International de Baillarguet, TA-A15/G, 34398 Montpellier, France; UMR 5290 Maladies Infectieuses & Vecteurs-Ecologie, Génétique, Ecologie, Contrôle (MIVEGEC), CNRS, Université de Montpellier, Montpellier, France; IRD, UR 224 MIVEGEC, Agropolis, BP 64501, 34 394 Montpellier cedex 5, France; INRA, UMR1309 CMAEE, 34398 Montpellier, France; EID Méditérranée, 34184 Montpellier, France; Istituto Zooprofilattico Sperimentale dell’Abruzzo e del Molise ‘G. Caporale’, 64100 Teramo, Italy

**Keywords:** *Culicoides*, Distribution range, Spatio-temporal, Population genetics, Entomological survey, Mediterranean basin

## Abstract

**Background:**

Introduction of vector species into new areas represents a main driver for the emergence and worldwide spread of vector-borne diseases. This poses a substantial threat to livestock economies and public health. *Culicoides imicola* Kieffer, a major vector species of economically important animal viruses, is described with an apparent range expansion in Europe where it has been recorded in south-eastern continental France, its known northern distribution edge. This questioned on further *C. imicola* population extension and establishment into new territories. Studying the spatio-temporal genetic variation of expanding populations can provide valuable information for the design of reliable models of future spread.

**Methods:**

Entomological surveys and population genetic approaches were used to assess the spatio-temporal population dynamics of *C. imicola* in France. Entomological surveys (2–3 consecutive years) were used to evaluate population abundances and local spread in continental France (28 sites in the Var department) and in Corsica (4 sites). We also genotyped at nine microsatellite loci insects from 3 locations in the Var department over 3 years (2008, 2010 and 2012) and from 6 locations in Corsica over 4 years (2002, 2008, 2010 and 2012).

**Results:**

Entomological surveys confirmed the establishment of *C. imicola* populations in Var department, but indicated low abundances and no apparent expansion there within the studied period. Higher population abundances were recorded in Corsica. Our genetic data suggested the absence of spatio-temporal genetic changes within each region but a significant increase of the genetic differentiation between Corsican and Var populations through time. The lack of intra-region population structure may result from strong gene flow among populations. We discussed the observed temporal variation between Corsica and Var as being the result of genetic drift following introduction, and/or the genetic characteristics of populations at their range edge.

**Conclusions:**

Our results suggest that local range expansion of *C. imicola* in continental France may be slowed by the low population abundances and unsuitable climatic and environmental conditions.

**Electronic supplementary material:**

The online version of this article (doi:10.1186/s13071-016-1426-4) contains supplementary material, which is available to authorized users.

## Background

Range expansions are increasingly frequent in the history of many, if not most, species [1]. The growing rate of such phenomena and the associated consequences could be terribly alarming when concerning arthropod vector species. Introduction of vector species into new areas can lead to the emergence and spread of human and animal vector-borne pathogens, posing substantial threat to public health and livestock economy. Although most range expansions are linked to anthropogenic influences such as intercontinental commerce and travel (e.g. ship, boat, aircraft, highways), species introductions can also occur by natural dispersal of the organisms or passive transport by winds (e.g. [2–4]). The dynamics of range expansion results from the interaction of ecological and evolutionary factors. These factors are most often analysed at a spatial scale while the temporal scale is overlooked. Assessing the drivers involved in range expansion can help in designing reliable predictive models of future spread. In particular, the study of spatio-temporal genetic variation of expanding populations can yield insights into their dispersion rates and patterns of spread, which in turn can prompt the processes underlying their establishment and persistence [5].

In this study, we investigated the spatio-temporal population dynamics of the biting midge *Culicoides imicola* Kieffer (Diptera: Ceratopogonidae) at its northern distributional edge in France. This species is known as the main afrotropical vector of Orbiviruses including bluetongue virus (BTV) and African horse sickness virus (AHSV) in Africa and the Middle-East [6]. *Culicoides imicola* was described as expanding its range northward in the Mediterranean basin, however, recent genetic studies suggest that the species has been present and established for a long time in the Mediterranean region [7, 8]. Nevertheless, if the settlement of *C. imicola* populations in most parts of the Mediterranean region is an old story [7], extensive entomological surveillance performed in continental France since 2002 following BTV emergence in Corsica Island suggests a recent northward expansion of the species at the northern edge of its distribution [9].

Following the record of *C. imicola* in the island of Sardinia (Italy), the species was collected for the first time in the French island of Corsica in 2000 [10]. These findings were confirmed by subsequent extensive entomological surveys undertaken from 2002 onwards [9, 11, 12]. Populations are found to be widely distributed throughout Corsica notably on the littoral zone, with high abundances (reaching 12,000 captured individuals per night during the first observations) [9], suggesting that *C. imicola* is well established in the island. Given the short distance between continental France and Corsica (180 km), a wide entomological surveillance network was implemented in 2002 in continental France along the Mediterranean coast to investigate the spread from Corsica. In 2003, the first *C. imicola* individuals were captured (two individuals) in south-eastern continental France, in Var and Alpes-Maritimes departments [9]. Despite subsequent additional surveys around the two positive sites, settled populations of *C. imicola* were not recorded [9]. In 2004, five more individuals were collected in the Var department; the next years witnessed records of other established populations of *C. imicola* [9]. The status of established population was based on the number of positive sites for at least two consecutive years with more than ten captured parous and/or blood-fed females [9]. The first collected individuals of *C. imicola* in the Var department coincided with a year of high abundance of the species in the north-eastern part of Corsica [9]. The authors hypothesized that Corsican emigrants of the species had most likely colonized the Var department through its high passive wind-mediated dispersal capacity. This was supported by genetic analyses based on microsatellite and mitochondrial markers which indicated strong genealogical relationships between populations of *C. imicola* from the Var department and Corsica [Jacquet S et al. Range expansion of the Bluetongue vector Culicoides imicola in continental France thanks to meteorological events (submitted)].

To date, the species distribution has reached its most northern distribution edge in the south-east of the Var department. Since the first records, population abundance is low in this zone (maximum catch < 200 individuals collected per night) [9]. In 2008, a new record of *C. imicola* in south-western continental France, in the littoral zone of the Pyrénées-Orientales department, confirmed the ability of this species to continue expanding its range and colonize new habitats [Jacquet S et al. Range expansion of the Bluetongue vector Culicoides imicola in continental France thanks to meteorological events (submitted)]. A combination of high population abundances and suitable wind dispersal capacity has been shown to contribute to the successful range expansion of this species [Jacquet S et al. Range expansion of the Bluetongue vector Culicoides imicola in continental France thanks to meteorological events (submitted)]. Besides, an ecoclimatic niche model predicted new suitable habitats under climate change for *C. imicola* populations in Europe [13].

In this paper, we further investigated the processes underlying the range expansion of *C. imicola* at a local scale in France. We updated the distribution and abundance of *C. imicola* in Corsica and Var department. We described the fine scale spatio-temporal genetic structuring of the populations of this vector species. Particularly, we focused on the levels of genetic diversity and differentiation between the range margin (Var department) and a more central distribution (Corsica and Sardinia) of *C. imicola*. The results were jointly interpreted to describe *C. imicola* population dynamics in these regions.

## Methods

### Entomological surveys

**Fig. 1:**
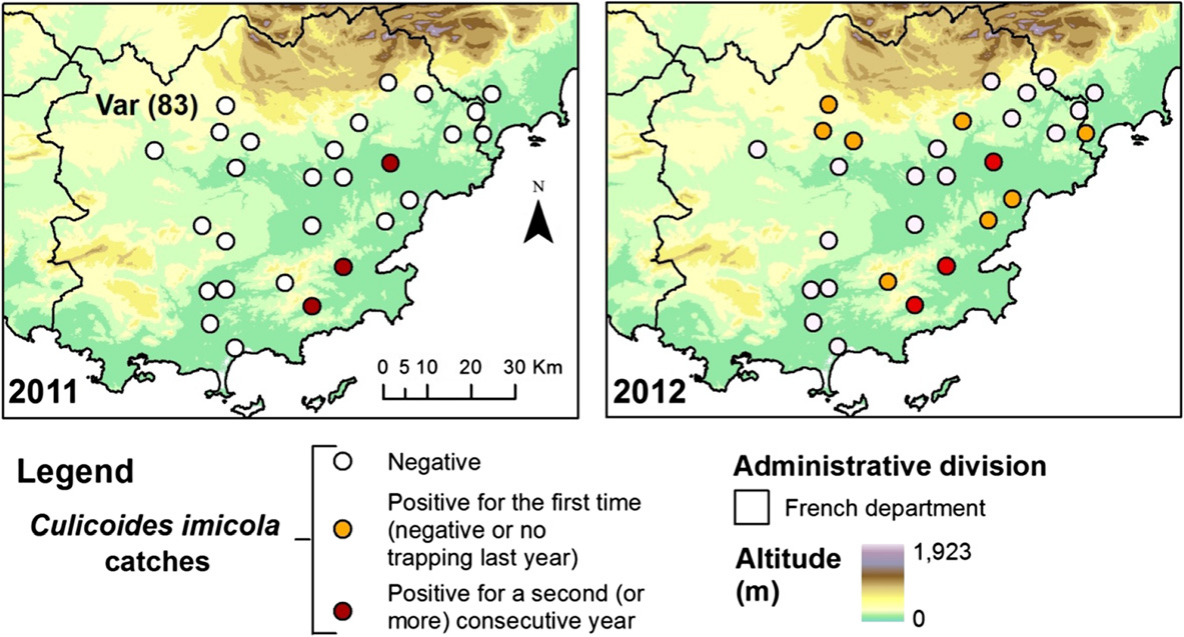
Distribution area of *C. imicola* in the Var department in 2011 and 2012. Presence/absence maps from 2003 to 2010 were previously published in [9]

**Fig. 2:**
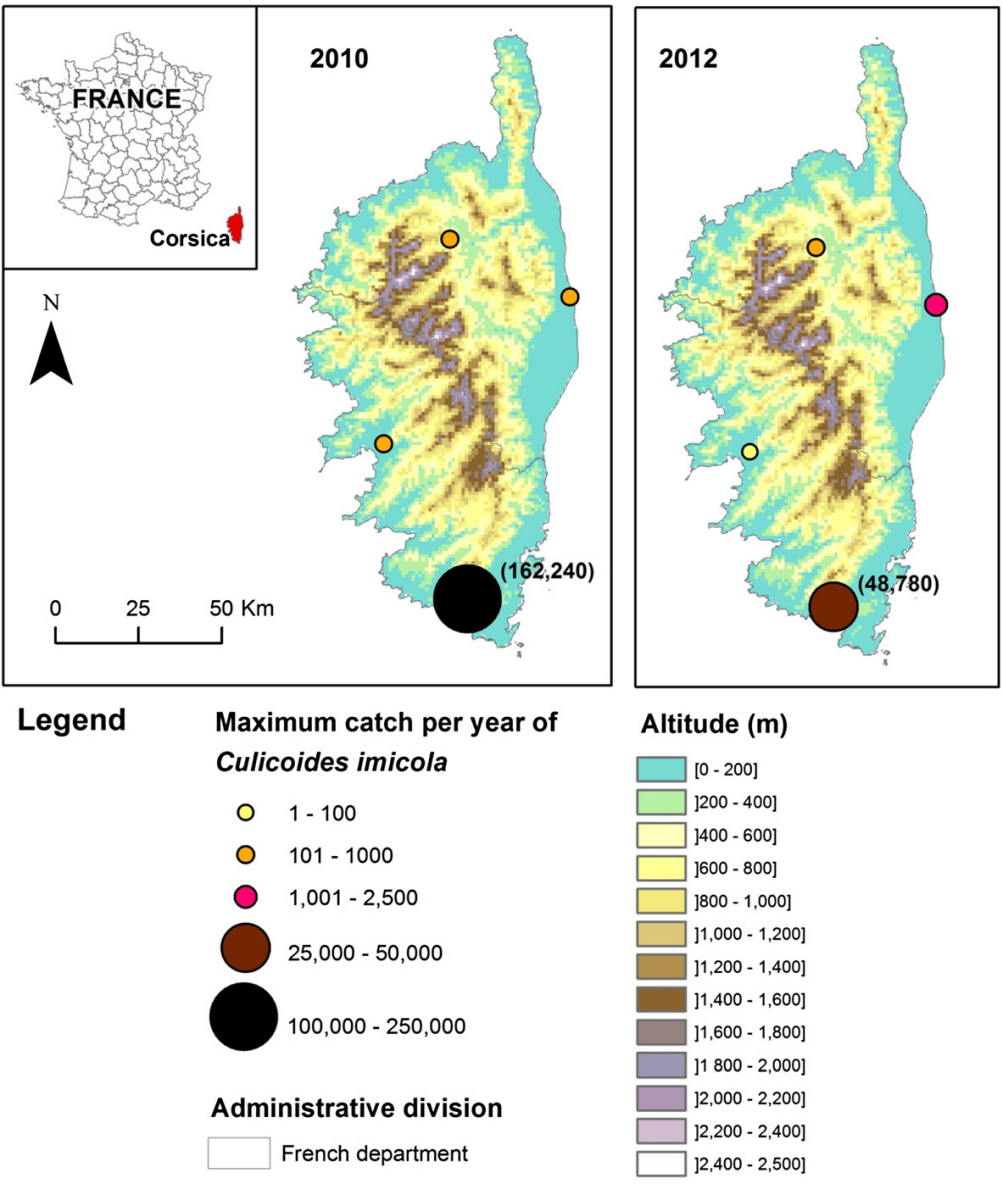
Collection sites in Corsica with maximum catches of *Culicoides imicola* per year in 2010 and 2012. Population abundance maps from 2003 to 2009 were previously published in [9]

To carry on the evaluation of settlement (presence/absence in sampled sites) and spatial expansion of *C. imicola* in Var and Alpes Maritimes departments, we continued the yearly surveillance network presented in [9]. Entomological surveys were carried out in twenty-eight and twenty-nine sites respectively in 2011 and 2012 (Fig. 1, Additional file 1: Table S2). Population abundance in the Var department was assessed for the period 2003–2014. Moreover, four sites were sampled in Corsica in 2010 and 2012 (Fig. 2, Additional file 1: Table S3) in order to survey population abundances. Most of these sites have been surveyed between 2003–2010 and 2002–2008 in the Var department and Corsica, respectively, in [9]. All sites in the Var department were sampled once a year during the end of summer/early autumn (September/October) to match the abundance peak of *C. imicola* [9]. In Corsica, sites were surveyed weekly (February-April, November-December) and monthly (January, May-October) with a single night collection.

### Population genetics collection

**Fig. 3:**
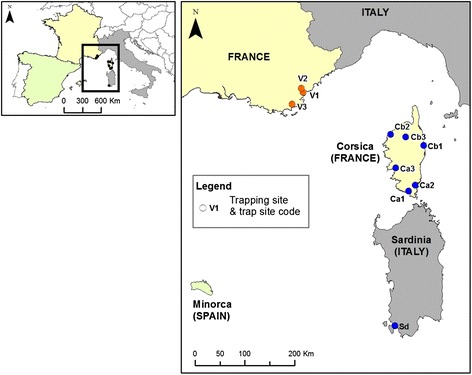
Sampled sites of *C. imicola* used for population genetic analyses

For genetic analyses, *C. imicola* samples collected from six sites over four years in Corsica (2002, 2008, 2010 and 2012), and from three sites over four years in the Var department (2006, 2008, 2010 and 2012) were selected (Fig. 3, Table 1). Genetic comparisons between Corsican and Var populations were conducted for the paired years. One location sampled in 2012 from Sardinia was also included (Table 1). Adult midges were caught using black-light suction traps (Onderstepoort design) placed near or inside animal shelters containing sheep, cattle or horses. Specimens were stored in 90 % ethanol and *C. imicola* individuals were identified and sexed under a binocular microscope using morphological determination keys [10].

**Table 1:** Geographical location of *C. imicola* sampled sites for the population genetics study

					2002		2006		2008		2010		2012	
Country	Locations	LON	LAT	Code	Collection date	N	Collection date	N	Collection date	N	Collection date	N	Collection date	N
France, Corsica	Figari	9.08	41.50	Ca1	10/09/2002	32	-	-	18/09/2008	28	07/09/2010	31	19/09/2012	32
France, Corsica	Porto-Vecchio	9.25	41.59	Ca2	10/09/2002	32	-	-	19/11/2008	32	-	-	-	-
France, Corsica	Bastelicaccia	8.82	41.94	Ca3	-	-	-	-	-	-	10/05/2010	31	13/11/2012	32
France, Corsica	San Giuliano	9.54	42.29	Cb1	12/11/2002	32	-	-	18/09/2008	32	07/09/2010	30	-	-
France, Corsica	Calvi	8.76	42.54	Cb2	28/10/2002	32	-	-	01/09/2008	32	-	-	-	-
France, Corsica	Moltifao	9.12	42.47	Cb3	-	-	-	-	26/08/2008	29	10/08/2010	31	20/11/2012	31
France, Var department	Roquebrune-sur-Argens	6.68	43.40	V1	-	-	20/06/2006	32	30/09/2008	32	10/09/2010	32	18/09/2012	32
France, Var department	Roquebrune-sur-Argens	6.64	43.49	V2	-	-	-	-	30/09/2008	32	10/09/2010	31	18/09/2012	30
France, Var department	Bormes-Les-Mimosas	6.40	43.20	V3	-	-	-	-	28/08/2008	27	-	-	20/09/2012	30
Italy, Sardinia	San Giovanni Suergiu	8.52	39.13	Sd	-	-	-	-	-	-	-	-	06/11/2012	32

N is the number of individuals typed for microsatellite analyses

The dashes indicate the absence of data

LAT and LON are the GPS coordinates, corresponding to the latitude and longitude, respectively

### Microsatellite genotyping

Genomic DNA was extracted from each single midge using the NucleoSpin96 Tissue Kit (Macherey-Nagel, Duren, Germany), according to the manufacturer’s instructions, starting with an additional step where each individual midge was ground in 200 μL of 1X PBS buffer. Each insect (~32 individuals per site) was genotyped at nine microsatellite markers previously developed for *C. imicola* [8] (Additional file 1: Table S1) following the protocol described in [7].

### Microsatellite analyses

#### Genetic diversity and equilibrium testing

Linkage disequilibrium between all pairs of loci and deviations from Hardy-Weinberg, i.e. significant deviation of the inbreeding coefficient F_IS_ from zero, were tested with FSTAT v2.9.3.2 [14]. The significance of F_IS_ was assessed by randomizing alleles among individuals within samples (10,000 permutations). Level of genetic diversity within samples per year was quantified by computing the allelic richness (A_R_) and the mean observed heterozygosity (H_O_) [15] with FSTAT v2.9.3.2 [14]. We used a non-parametric Mann-Whitney-Wilcoxon statistical test in R software v.3.1.2 [16] to check for any differences in levels of allelic richness between Corsican and Var populations.

To test whether populations are in mutation-equilibrium, we performed tests of heterozygosity excess [17] implemented in BOTTLENECK v.1.2.02 [18]. It has been shown that past bottleneck events will be detected with a high degree of sensitivity using the Infinite Allele Mutation (IAM) model, moderately with the Two-phase Model (TMM) and dimly with the Stepwise Mutation Model (SMM) [17]. Heterozygosity excess was tested under all three mutation models. For the TPM model the proportion of SMM was set to 70 % and the variance to 30 (default values). The significance was assessed by performing 10,000 replicates. The Wilcoxon’s signed-rank statistics were used to evaluate any deviation of the observed heterozygosity from the expectation under mutation-drift equilibrium.

#### Population structure

Genetic relationships between samples were visualized by a neighbor-joining tree (NJ) tree [19] based on the pairwise genetic distances of Cavalli-Sforza and Edwards [20] using the software POPULATIONS v.1.2.30 (http://bioinformatics.org/~tryphon/populations/). The robustness of the tree topology was evaluated by bootstrapping 10,000 times over loci.

Genetic variation and population structuring was also investigated by undertaking a Principal Component Analysis (PCA) using PCA-GEN v.1.2.1 software [21]. This analysis correlates genotypes and allele frequencies among individuals, without any assumption of equilibrium within populations, to define variables (components) that can characterize the neutral genetic variation among populations. The statistical significance associated with each axis was evaluated after 10,000 randomizations.

The genetic structure of *C. imicola* was then assessed using the Bayesian clustering method implemented in STRUCTURE v.2.3.3 software [22] which ascertains population membership of individuals according to their genotypes. We assumed an admixture model and correlated allele frequencies and used the sampling location as prior information. Each run consisted of a burn-in of 10^5^ Markov chain Monte Carlo (MCMC) iterations, followed by 10^6^ iterations. Ten replicates were carried out for each number K of genetic clusters set between 1 and 9. The accurate number of clusters was inferred with the ∆K method [23]. The relative importance of the genetic clusters previously inferred by STRUCTURE and the population differentiation was assessed with the multi-locus hierarchical F-statistics. We tested if the genetic patterns were explained by the geography and/or the collection dates by grouping hierarchically the samples according to their origin (Corsica *vs* Var department), then their collection year. We then performed the test separately within Corsica and Var departments: samples were grouped hierarchically according to their collection year to assess any genetic significant differentiation during the time studied. This analysis was performed with Hierfstat package [24]. These tests were based on 10,000 permutations of either *Culicoides* genotypes among populations, within groups (i.e. collection dates) and within clusters (i.e. Corsica and Var department; H0: ‘Fpopulations-cluster = 0’), or populations among clusters (H0: ‘Fclusters-total = 0’).

The software FSTAT v2.9.3.2 was used for assessing the level of genetic differentiation among populations through the Weir and Cockerham [25]’s unbiased estimates of F_ST_*.*

These analyses were performed per year over the total sampling set available (encompassing thus Corsica and Var). A significant deviation of F_ST_ from zero was tested using the exact G test over 10,000 permutations of genotypes among samples. Global F_ST_ value was estimated over all sampled sites (Corsica and Var) in either 2008 or 2012 (i.e., two years when the sampling sets included the exact same sampled sites). A statistical non-parametric Wilcoxon signed test with continuity correction implemented in R software v.3.1.2 [16] was used to test whether the genetic differentiation between Corsican and Var populations has significantly increased over time. Specifically we tested if the genetic differentiation (based on F_ST_ values) in 2012 was significantly higher than in 2008.

#### Isolation by distance

To test whether patterns of neutral genetic structure of *C. imicola* Corsican and Var populations were related to geographic distance, we performed partial Mantel tests implemented in the online program GENEPOP v.4.2 [26, 27]. These tests were conducted separately for the years 2008 and 2012. The significance of the regression between the logarithmic geographic distances and the pairwise values of F_ST_ / (1 - F_ST_) was assessed with 100,000 permutations.

## Results

### Entomological surveys

**Fig. 4:**
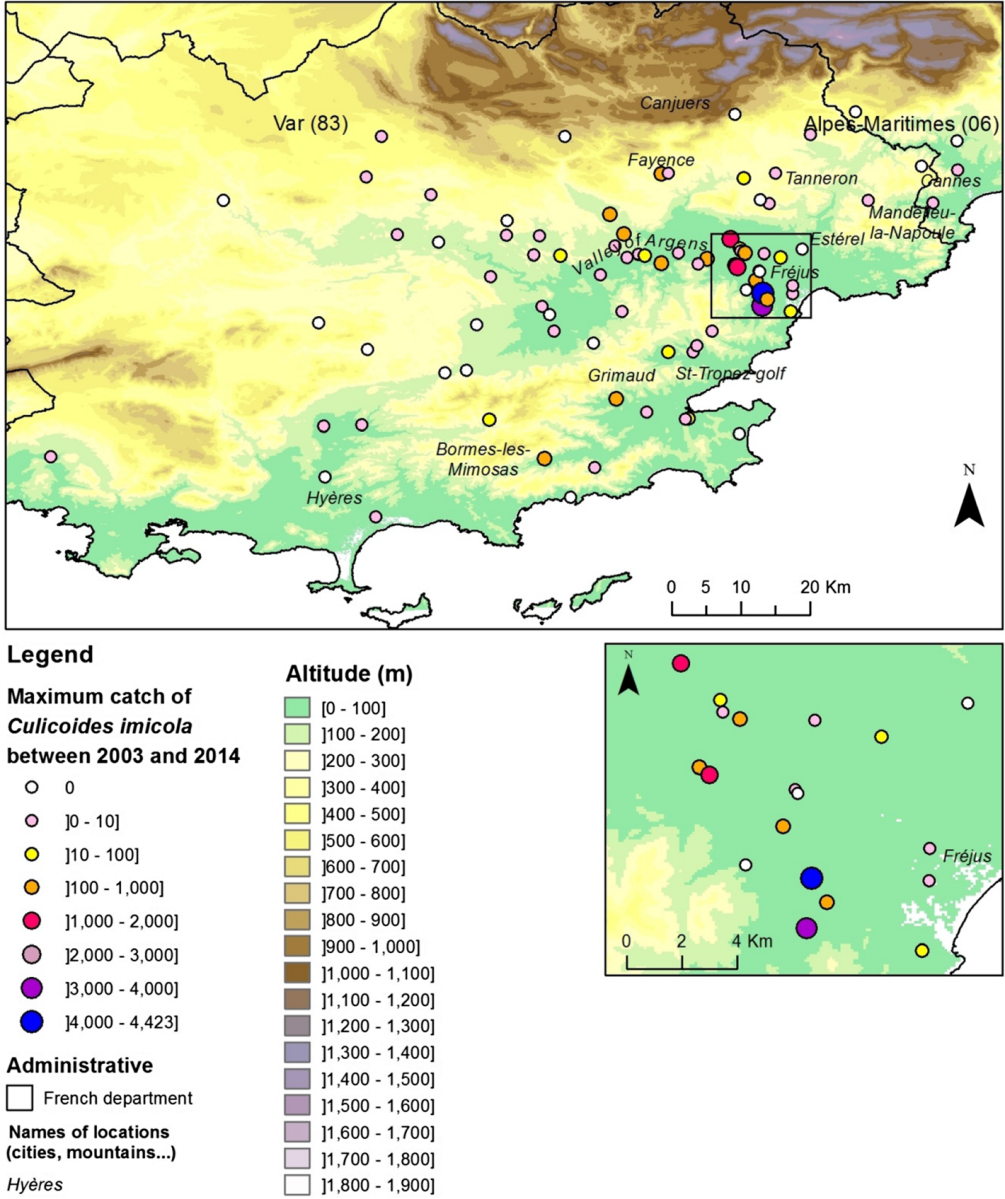
Maximum catch per year of *C. imicola* in the Var department from 2003 to 2014

A total of twenty-eight and twenty-nine sites were surveyed in 2011 and 2012 respectively (Additional file 1: Table S2). In 2011, three out of the twenty-eight sites studied were previously recorded positive in [9] and were positive for a second consecutive year (Fig. 1). In 2012, eight new positive sites were recorded (Fig. 1). The compiled maximum catch per year of *C. imicola* between 2003 and 2014 showed low abundances in the Var department since the maximum did not exceed 4,500 individuals per site and night (Fig. 4). In Corsica, *C. imicola* population abundances were always high in the most southern site, as it was recorded before in [9], reaching a maximum of about 160,000 *C. imicola* caught per night in 2010. The three other sites exhibited relatively low abundance, never exceeding 2,500 individuals caught per night for the three studied years (Fig. 2).

For genetic analyses, a total of 809 individuals from six sites sampled over four years (2002, 2008, 2010 and 2012) in Corsica and from three sites collected over four years (2006, 2008, 2010 and 2012) in the Var department were genotyped and analysed (27–32 individuals per site; Table 1).

### Microsatellite analyses

#### Within population genetic diversity through time

Pairwise allelic tests of linkage disequilibrium indicated that loci were physically unlinked and statistically independent within populations (*P*-value > 0.0013 after Bonferroni correction). Fisher’s exact test revealed that genotypic frequencies were in accordance with Hardy-Weinberg equilibrium for all populations (*P*-value > 0.0002 after Bonferroni correction), F_IS_ values ranged from −0.075 to 0.140 (Table 2).

**Table 2:** Genetic diversity and bottleneck results based on microsatellite data for each sampled site

Country	Locations	Code	A_R_	H_O_	H_E_	F_IS_	*P*-values	IAM	TPM	SMM
France-Corsica	Figari	Ca1_02	4.390 ± 1.311	0.534 ± 0.132	0.569 ± 0.100	0.061	0.0961	0.082	0.633	0.981
France-Corsica	Figari	Ca1_08	4.483 ± 1.583	0.573 ± 0.156	0.594 ± 0.121	0.037	0.1998	**0.010**	0.285	0.850
France-Corsica	Figari	Ca1_10	4.285 ± 1.188	0.582 ± 0.154	0.590 ± 0.141	0.014	0.3648	**0.005**	**0.024**	0.935
France-Corsica	Figari	Ca1_12	4.263 ± 1.310	0.574 ± 0.144	0.592 ± 0.125	0.031	0.2372	**0.019**	0.150	0.590
France-Corsica	Porto-Vecchio	Ca2_02	4.541 ± 1.481	0.552 ± 0.112	0.572 ± 0.104	0.034	0.2074	0.082	0.633	0.976
France-Corsica	Porto-Vecchio	Ca2_08	4.431 ± 1.512	0.555 ± 0.110	0.599 ± 0.095	0.074	0.0440	**0.010**	0.180	0.850
France-Corsica	Bastelicaccia	Ca3_10	4.562 ± 1.688	0.616 ± 0.218	0.603 ± 0.138	−0.022	0.6934	**0.014**	0.367	0.820
France-Corsica	Bastelicaccia	Ca3_12	4.568 ± 1.365	0.549 ± 0.182	0.594 ± 0.159	0.077	0.0257	**0.019**	0.082	0.976
France-Corsica	San Giuliano	Cb1_02	4.240 ± 0.999	0.566 ± 0.154	0.574 ± 0.129	0.014	0.4029	**0.010**	0.367	0.850
France-Corsica	San Giuliano	Cb1_08	4.476 ± 1.194	0.661 ± 0.117	0.616 ± 0.087	−0.075	0.9621	**0.010**	0.285	0.787
France-Corsica	San Giuliano	Cb1_10	4.293 ± 1.369	0.550 ± 0.177	0.578 ± 0.116	0.050	0.1200	**0.010**	0.326	0.850
France-Corsica	Calvi	Cb2_02	4.368 ± 1.327	0.601 ± 0.150	0.611 ± 0.110	0.018	0.3430	**0.007**	0.180	0.674
France-Corsica	Calvi	Cb2_08	4.264 ± 0.979	0.523 ± 0.134	0.607 ± 0.115	0.140	0.0040	**0.002**	0.150	0.820
France-Corsica	Moltifao	Cb3_08	4.219 ± 1.358	0.546 ± 0.120	0.603 ± 0.110	0.096	0.0136	**0.001**	0.064	0.752
France-Corsica	Moltifao	Cb3_10	4.121 ± 0.867	0.611 ± 0.162	0.605 ± 0.131	−0.010	0.5996	**0.005**	0.064	0.590
France-Corsica	Moltifao	Cb3_12	4.307 ± 1.330	0.568 ± 0.197	0.576 ± 0.154	0.014	0.3401	0.064	0.410	0.850
France-Var department	Roquebrune-sur-Argens	V1_06	3.680 ± 0.794	0.560 ± 0.100	0.561 ± 0.082	0.103	0.0084	**0.005**	**0.020**	0.590
France-Var department	Roquebrune-sur-Argens	V1_08	3.501 ± 0.926	0.609 ± 0.112	0.584 ± 0.094	−0.044	0.4916	**0.002**	**0.010**	0.326
France-Var department	Roquebrune-sur-Argens	V1_10	3.690 ± 1.366	0.603 ± 0.150	0.587 ± 0.100	−0.029	0.8393	**0.001**	**0.005**	0.213
France-Var department	Roquebrune-sur-Argens	V1_12	3.566 ± 1.176	0.573 ± 0.125	0.592 ± 0.091	0.033	0.7551	**0.001**	**0.001**	0.082
France-Var department	Roquebrune-sur-Argens	V2_08	3.525 ± 0.361	0.515 ± 0.131	0.556 ± 0.127	0.075	0.2253	**0.003**	0.064	0.545
France-Var department	Roquebrune-sur-Argens	V2_10	3.744 ± 1.091	0.596 ± 0.146	0.581 ± 0.106	−0.027	0.0492	**0.003**	**0.014**	0.367
France-Var department	Roquebrune-sur-Argens	V2_12	4.031 ± 0.957	0.591 ± 0.103	0.570 ± 0.093	−0.038	0.8049	**0.005**	0.285	0.875
France-Var department	Bormes-Les-Mimosas	V3_08	3.820 ± 1.045	0.558 ± 0.147	0.572 ± 0.122	0.024	0.3183	**0.014**	0.150	0.633
France-Var department	Bormes-Les-Mimosas	V3_12	4.067 ± 1.045	0.519 ± 0.160	0.558 ± 0.117	0.072	0.0619	**0.005**	0.410	0.981
Italy-Sardinia	San Giovanni Suergiu	Sd_12	4.513 ± 1.926	0.560 ± 0.156	0.590 ± 0.143	0.052	0.0868	**0.018**	0.248	0.787

The allelic richness (A_R_), observed (H_O_) and expected (H_E_) heterozygosity and F_IS_ are presented for each population. A_R_ is based on the minimum sample size of 27 diploid individuals. F_IS_ *P*-value adjusted at the nominal level (5 %) is 0.0002 after Bonferroni correction. Results of bottleneck tests (*P*-values) are presented for the Infinite Allele Model (IAM), Two-phase Model (TPM) and Stepwise Mutation Model (SMM)

The number at the end of each sample code corresponds to the collection year

Significant results of bottleneck tests are indicated in bold

Levels of genetic diversity remained stable over populations and over years (Table 2). In Corsica, the allelic richness (A_R_) varied from 4.188 to 4.725 and the mean heterozygosity (H_O_) ranged from 0.523 to 0.616. Both measures were comparable in Sardinia with an allelic richness equal to 4.512 and a mean heterozygosity of 0.560. In the Var department, the allelic richness extended from 3.481 to 4.214 and the mean observed heterozygosity varied from 0.515 to 0.609. Interestingly, the allelic richness was statistically lower in the Var department than in Corsica (*P* < 0.0001).

#### Spatio-temporal genetic changes in population structure

**Fig. 5:**
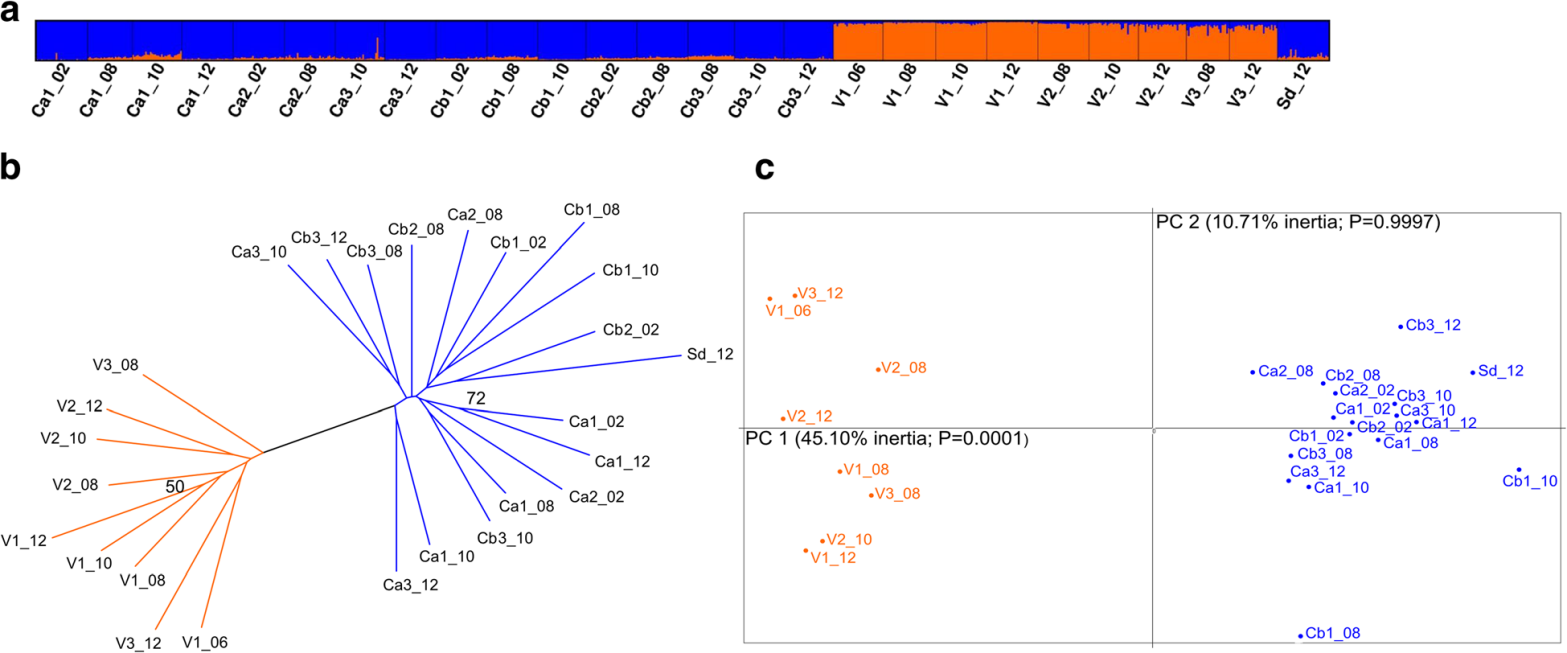
Population genetic structure results of *Culicoides imicola*. **a** Genetic clustering of *C. imicola* samples. Each vertical line represents an individual and each colour represents a cluster. **b** Microsatellite neighbor-joining tree based on genetic distance of Cavalli-Sforza & Edwards (1967). Bootstrap values are calculated over 1,000 replicates (only values > 50 % are shown). **c** Principal Component Analysis based on microsatellite allelic frequencies

A spatial genetic structure was detected between, on the one hand, the group formed of Corsican and Sardinian populations, and on the other hand, the continental populations from the Var department. The Bayesian clustering analysis defined these two geographical groups (∆K maximum for K = 2) (Fig. 5a). The same pattern was also evidenced in the neighbor-joining tree and the Principal Component Analysis (PCA) which grouped the sampled sites according to their geographical origin, without any effect of the collection year (Fig. 5b and c). The PCA showed a significant overall F_ST_ value of 0.034, and indicated that the first two axes were significant and explained 55.81 % of the total inertia: axis 1 accounted for 45.10 % of the variance (F_ST_ = 0.016, *P*-value = 0) and axis 2 accounted for 10.71 % of the variance (F_ST_ = 0.004, *P*-value = 0).

Considering the hierarchy of sampling, significant differentiation was detected for all levels: between the two genetic clusters Corsica and Var (Fclusters-total = 0.0021; *P* = 0.0085), within clusters (i.e. between collection years; Fyears-clusters = 0.0024; *P* = 0.0001) and within group (i.e. between samples collected at the same year; Fpopulations-years = 0.0247; *P* = 0.0022). When performing the analysis within Corsica, the collection year did not significantly account for the observed pattern (i.e. no significant difference between collection year; Fyears-total = 0.0032; *P* = 0.9755), however, significant differentiation was detected among samples for each year (Fpopulations-years = 0.0343; *P* = 0.0002). The same results were obtained for Var department (Fyears-total = 0.0079; *P* = 0.6877 and Fpopulations-years = 0.0071; *P* = 0.0021).

These results are globally consistent with the genetic differentiation tests, which indicated significant genetic differentiation between island and continental populations (Additional file 1: Table S4). Such differentiation is likely to have increased over time as indicated by the F_ST_ values which are twice as high in 2012 compared to 2008 (Table 3) and the significant Wilcoxon signed rank test (*P* = 0.0122). This was also evident in the estimates of global F_ST_ over all populations per year: values were higher in 2012 (F_ST_ = 0.032, *P* -value = 0.0002) than 2008 (F_ST_ = 0.014, *P* -value = 0.0001). Interestingly, populations from Sardinia were significantly differentiated to those from the Var department but were not differentiated to the Corsican populations (3 significant values over 16 comparisons; Additional file 1: Table S4).

**Table 3:** Pairwise F_ST_ values between Corsican and Var *C. imicola* population samples

	Cb3_08	V1_08	V2_08	V3_08
Ca1_08	−0.0076 (0.785)	**0.0247** (0.005)	**0.0251** (0.005)	**0.0259** (0.005)
Cb3_08		**0.0240** (0.005)	**0.0138** (0.005)	**0.0144** (0.005)
V1_08			0.0034 (0.670)	0.0089 (0.010)
V2_08				0.0047 (0.320)
	Cb3_12	V1_12	V2_12	V3_12
Ca1_12	0.0027 (0.030)	**0.0506** (0.005)	**0.0400** (0.005)	**0.0431** (0.005)
Cb3_12		**0.0501** (0.005)	**0.0457** (0.005)	**0.0385** (0.005)
V1_12			**0.0139** (0.005)	0.0206 (0.035)
V2_12				−0.0004 (0.095)

Pairwise F_ST_ values were computed for 2008 (atop) and 2012 (below). Significant F_ST_ are represented in bold. *P*-values are indicated in brackets, indicative adjusted nominal level (5 %) for multiple comparisons after Bonferroni correction is 0.005

Assessing the levels of genetic differentiation over all collected years within regions indicated that sampled populations within Corsica and the Var department were not markedly structured. Indeed, the computed F_ST_ values were relatively low, ranging respectively from −0.0104 to 0.0290 in Corsica and −0.0029 to 0.0261, in the Var department (Additional file 1: Table S4). None of the pairwise comparisons was significant within Corsica (*P* -value > 0.0042), and only four out of the thirty-six comparisons within the Var department were significant (*P* -value < 0.0014, after Bonferonni correction). These results did not support any evident changes in genetic differentiation within each region during the time period studied.

#### Equilibrium testing

While tests based on the IAM mutation model suggested potential signatures of past genetic bottlenecks in nearly all sampled sites over years, those based on the most realistic TPM and SMM mutation models were only significant for one Corsican site sampled in 2010 (Figari) and two sites in the Var department, including Roquebrune-sur-Argens (V1, over the four collection years and V2 in 2010) (Table 2).

#### Isolation by distance

**Fig. 6:**
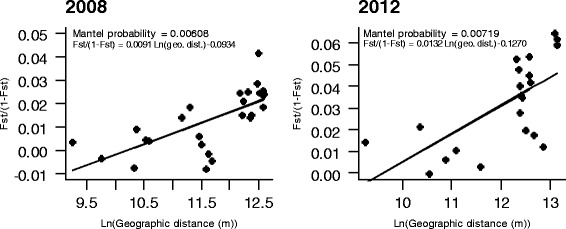
Results of the Mantel tests for isolation by distance (IBD)

Patterns of isolation by distance among *C. imicola* sampled populations were observed both in 2008 and in 2012 (*P* -value = 0.00608 and 0.00719, respectively; Fig. 6).

## Discussion

While a recent study claims the historical presence of *C. imicola* populations in the Mediterranean region [7], continuous northward expansion of this vector species has been predicted as a result of projected climate change in the future [13]. Climate changes during the Pleistocene would have opened new suitable habitats and allowed the northward expansion of the species from northern sub-Saharan Africa to the North African coast [7]. Nevertheless, if *C. imicola* is historically present in these regions, entomological surveys suggest recent expansion of settled populations at the northern edge of the species distribution. Particularly, introduction of the species in the Var department in continental France, through wind-mediated dispersal from Corsica, highlights the potential of *C. imicola* to continue expanding its range and colonize new habitats. In this study, we investigated the spatio-temporal patterns of genetic variation and population dynamics of *C. imicola* at a local scale in a recent colonized area a priori, i.e. the Var department, and a region where the species has been established for a longer time, i.e. Corsica. Using nine highly polymorphic markers, two main conclusions can be drawn from our results: (i) the genetic composition and diversity within region did not significantly change in the time period studied and (ii) the genetic differentiation among Var and Corsican populations has significantly increased when considering the years when sampling had been paired between the two regions.

Our results suggest that the genetic diversity as well as the genetic structure within Corsica and the Var department have remained relatively stable during the time period studied. Indeed, the principal component analysis indicated that virtually none of the variation could be attributed to temporal samples within regions, but was explained by spatial differentiation between regions. The observed low population structure within regions is an expected response to considerable gene flow between populations. Species from the genus of *Culicoides* are described as weak active flyers because of their small size. The daily flight distance for European *Culicoides* species in the Palearctic region has been estimated to be up to 2.21 km [28, 29], but is still unknown for *C. imicola*. In the Var department, the average spread of *C. imicola* has been estimated to be 14.5 km/year, suggesting a limited local expansion of the species [9]. The limited density of *C. imicola*, the presence of physical barriers and potential unsuitable environmental conditions probably impair its ability to successfully disperse [9]. In contrast, our findings indicate that *C. imicola* may passively and/or actively disperse at the local scale, allowing relatively high gene flow among populations. It is worth noting that the number of sampled sites in the Var department was small and that the sites were geographically close to each other, and thus may not allow an accurate determination of the genetic structuring. However, despite the hilly topography of Corsica and the geographical distances between the sampled sites, low genetic differentiation was also observed among Corsican populations supporting the existence of gene flow among them. Nevertheless, it is possible that our set of markers have failed to assess the population structure at this scale. It would thus be interesting to further investigate the fine-scale spatio-temporal population variation with more markers or by performing genomic analysis based on highly polymorphic markers such as single nucleotide polymorphisms (SNPs). This would allow for assessing the impact of landscape on population structuring. It would also be interesting to estimate the spread of *C. imicola* Corsican populations that are more abundant, using direct methods in order to evaluate the dispersal ability of the species in the landscape.

A significant increase in the genetic differentiation between Var and Corsican populations was observed through time. This genetic pattern could be explained by two hypotheses. First, it could reflect the genetic changes in population structuring due to founder effects following introduction. Indeed, incursion of species in novel environments is often associated with a loss of genetic diversity when the gene pool in the new habitat is provided by a small number of founding individuals [30]. The newly founded population may then experience strong genetic drift resulting in genetic differentiation among populations [30]. Our results support this scenario since signatures of demographic bottlenecks were detected in two Var populations and one Corsican population. In addition, the levels of allelic richness were significantly lower in the Var department, while the mean heterozygosity was comparable among all populations. These results are consistent with the loss of genetic diversity associated with founder effects, as allelic richness is expected to be more sensitive to the effects of bottlenecks than is heterozygosity [30–32]. Thus, a limited number of emigrant midges carried by the wind from Corsica Island into the Var department could have been the target of genetic drift, leading to the observed genetic differentiation.

Complementarily, the observed patterns could result from the processes operating at the distributional edge of *C. imicola* populations. Indeed, it is widely appreciated that towards the range edge, habitats and ecological factors can influence the demography of populations, leading to habitat fragmentation and low population abundance and density [1, 33]. As a result, genetic diversity is likely to decrease while genetic differentiation is expected to increase in marginal populations compared with central populations [33]. Such patterns have been reported for many taxa including plants (e.g. [34, 35]), insects (e.g. [36]) and reptiles (e.g. [37]). Despite regular entomological surveys, *C. imicola* populations have not been found in neighbouring areas of the south-east coastal region of the Var department, its present consensually admitted northern edge [9]. In addition, levels of *C. imicola* abundance in this region have remained very low since its first records (maximum of 4,500 individuals caught per night over 2003–2014). Using similar trapping protocols (i.e. black-light suction traps placed in the near vicinity of animal shelters containing sheep, cattle or horses), other reported local abundance such as in Corsica (maximum catch of ~ 160,000 in 2010) [9] and Sardinia (10,000-65,000 maximum catch/night) [38] were about ten to forty times higher. Interestingly, lower levels of *C. imicola* abundance have also been reported in northern Spain, i.e. Catalonia, Basque country (<1,000 maximum catch/night) [39, 40] and northern continental Italy, i.e. north of Tuscany (<100 maximum catch/night) [38], marking respectively the north-western and north-eastern limits of the species distribution in the western Mediterranean area (the population recorded in south-western Continental France in the Pyrénées-Orientales department is still under investigation to assess the establishment of the population [Jacquet S et al.: Range expansion of the Bluetongue vector Culicoides imicola in continental France thanks to meteorological events (submitted)]). Although different types of trap have been used in these studies (i.e. Mini CDC trap in Spain and Onderstepoort trap in the Var department and Italy), previous trap comparison experiments have showed relatively small abundance differences between these traps [41, 42]. It thus appears that the observed low abundance levels at the northern edge of the species distribution are unlikely due to trapping methodology, but instead may reflect the role of abiotic and/or biotic factors in *C. imicol*a distribution. The patchy distribution and low population abundance of *C. imicola* populations are most likely driven by local habitat conditions including climate, topography, soil (type and moisture) and host availability [6, 43, 44], of which the species is highly dependent. Thus, environmental conditions in the Var department may be less suitable for *C. imicola* than in southern Corsica for example. As a consequence, the resulting low population size in the Var department may be more subject to demographic stochastic events and genetic drift. This may explain the observed genetic differentiation between Corsican and Var populations; however, we would also expect higher genetic differentiation between Var populations compared with Corsican populations. Yet, our results indicate low levels of genetic differentiation within Var. These low levels of differentiation could simply result from a recent introduction of *C. imicola* there; if the incursion of the species has occurred in 2004, populations may not have had enough time for a significant differentiation. In this context, the observed genetic patterns (i.e. signature of genetic bottleneck and lower genetic diversity and genetic differentiation in the Var department) and population abundances may support the hypothesis of a recent introduction in the Var department as suggested by entomological surveys [9], but a longtime presence of *C. imicola* in Corsica as highlighted by [7].

Interestingly, Corsican and Sardinian populations were genetically similar. The high capacity of *C. imicola* to passively disperse by winds may allow high gene flow among these two nearby islands, as it has been evidenced on longer distances in the Mediterranean region [Jacquet S et al.: Range expansion of the Bluetongue vector Culicoides imicola in continental France thanks to meteorological events (submitted)]. In contrast, the increase of genetic differentiation between Var and Corsican populations could indicate that despite the high dispersal ability of the species, migrations between Corsica and the Var department, if there are at all, may not be sufficient to homogenize the genetic composition of the continental populations. Nonetheless, these findings should be further investigated and confirmed by increasing the time period studied.

## Conclusion

Our study highlights the processes underlying contemporary range expansions and driving population dynamics at a local scale. Our results suggest that local range expansion of *C. imicola* in continental France may be slowed by low population abundances and unsuitable environmental conditions. Despite the high ability of *C. imicola* to passively disperse through winds, our results indicate that the presence of large water bodies may restrict this process allowing the genetic differentiation between Corsican and Var populations. Further analyses based on a wider temporal scale would help for a better understanding of the mechanisms shaping *C. imicola* distribution and therefore assessing local *C. imicola*-borne disease epidemiology. Nevertheless, our findings yield information for the design of predictive models of future spread.

## Additional file

Additional file 1: Table S1. Primers used for the amplification of the microsatellite loci in *C. imicola* (Mardulyn *et al*. 2013). **Table S2.** Details of the entomological surveys realized in Var department in 2011 and 2012. **Table S3.** Details of the entomological surveys realized in Corsica in 2010 and 2012. **Table S4.** Pairwise F_ST_ values between Corsican and Var populations of *C. imicola* for all collected years. (DOCX 58 kb)
