# Supplementary material for: Spatio-temporal genetic variation of the biting midge vector species Culicoides imicola (Ceratopogonidae) Kieffer in France
Source: Parasit Vectors. 2016 Mar 11;9:141. doi: 10.1186/s13071-016-1426-4 (PMC4788842; doi:10.1186/s13071-016-1426-4)
Supplement: Additional file 1: Table S1. — Primers used for the amplification of the microsatellite loci in C. imicola (Mardulyn et al. 2013). Table S2. Details of the entomological surveys realized in Var department in 2011 and 2012. Table S3. Details of the entomological surveys realized in Corsica in 2010 and 2012. Table S4. Pairwise FST values between Corsican and Var populations of C. imicola for all collected years. (DOCX 58 kb) [file 13071_2016_1426_MOESM1_ESM.docx]

**Additional file**

**Table S1 Primers used for the amplification of the microsatellite loci in *C. imicola* (Mardulyn *et al*. 2013).**

| **Loci** | **Motif** | **Forward / Reverse** | **Allele size range (bp)** | **Ta (°C)** |
| --- | --- | --- | --- | --- |
| 68 | (GT)n | CTTTTCCGTTTCTTTTTATTTCTTT | 101-105 | 60 |
|  |  | GTTTCTTTCTGGTCGCGTTGGTTGCTG |  |  |
| 12b | (CT)n | TTATGTGTGTATGTTAGCAAGGTCA | 133-139 | 50 |
|  |  | GTTTCTTCTTCGGATCAAAGAAATTTTGCC |  |  |
| 3b | (AC)n | ATGCGGATGTTTGAAGTG | 154-175 | 50 |
|  |  | GTTTCTTTTTTGTGTCTTATTGCCC |  |  |
| 31 | (CAA)n | TTCTGTTCGGCTGTTGCGTT | 162-166 | 60 |
|  |  | GTTTCTTCTTTTTACGTGGTGGTCATTC |  |  |
| 41b | (CT)n | GAGGAGGAGGTAGAA | 162-166 | 50 |
|  |  | GTTTCTTTCTATTAGTCAATGGTG |  |  |
| 35t | (AC)n | TTTGTAAAAGCCAGTTCAACCG | 181-188 | 60 |
|  |  | GTTTCTTATCGAACGAAGGAAATAACCAC |  |  |
| 88b | (AC)n | TTTGTTCGATTTGTAGTG | 243-256 | 50 |
|  |  | GTTTCTTCCTCTCTTTCATTCGC |  |  |
| 16 | (TG)n | TTGCCTTTGCTTGTTGAGGATG | 292-299 | 60 |
|  |  | GTTTCTTTCCTCTTTAAAATCACTGACGTG |  |  |
| 88 | (CAT)n | GTTGGTGCTTTGTTGTGTTGT | 344-348 | 50 |
|  |  | GTTTCTTTTTCTTTTTCTCCTTTTTGTTTCTTTC |  |  |

**Table S2 Details of the entomological surveys realized in Var department in 2011 and 2012.**

|  |  | 2011 | | 2012 | |
| --- | --- | --- | --- | --- | --- |
| Code site | Location | Collection date | Nb of *C. imicola* collected per trap per night | Collection  Date | Nb of *C. imicola* collected per trap per night |
| 06BT0 | Mandelieu-La Napoule | 04/10/11 | 0 | 18/09/12 | 1 |
| 06BT1 | Saint-Cézaire-sur-Siagne | - | - | 19/09/12 | 0 |
| 06BT03 | Pégomas | 03/10/11 | 0 | 18/09/12 | 0 |
| 83BT01 | Roquebrune-sur-Argens | 05/10/11 | 11 | 17/09/12 | 134 |
| 83BT16 | Grimaud | 05/10/11 | 7 | 19/09/12 | 52 |
| 83BT31 | Sainte-Maxime | 05/10/11 | 0 | 19/09/12 | 9 |
| 83BT33 | Bormes-Les-Mimosas | 04/10/11 | 27 | 18/09/12 | 701 |
| 83BT35 | Hyères | 04/10/11 | 0 | 18/09/12 | 0 |
| 83BT40 | Taradeau | 05/10/11 | 0 | 17/09/12 | 0 |
| 83BT43 | Les Adrets-de-L’Estérel | 03/10/11 | 0 | 18/09/12 | 0 |
| 83BT44 | Tanneron | 03/10/11 | 0 | 18/09/12 | 0 |
| 83BT46 | Saint-Paul-en-Forêt | - | - | 20/09/12 | 0 |
| 83BT50 | Draguignan | 05/10/11 | 0 | 20/09/12 | 0 |
| 83BT56 | Entrecasteaux | 06/10/11 | 0 | 20/09/12 | 0 |
| 83BT57 | Le Cannet-des-Maures | 05/10/11 | 0 | 19/09/12 | 0 |
| 83BT64 | Cabasse | 03/10/11 | 0 | 17/09/12 | 0 |
| 83BT66 | Collobrières | 04/10/11 | 0 | 18/09/12 | 25 |
| 83BT69 | Besse-sur-Issole | 03/10/11 | 0 | 17/09/12 | 0 |
| 83BT70 | Camps-la-Source | 03/10/11 | 0 | - | - |
| 83BT71 | Cuers | 04/10/11 | 0 | 20/09/12 | 0 |
| 83BT72 | Salernes | 06/10/11 | 0 | 20/09/12 | 1 |
| 83BT74 | Seillans | 04/10/11 | 0 | 20/09/12 | 0 |
| 83BT75 | Callian | 04/10/11 | 0 | 19/09/12 | 0 |
| 83BT76 | Aups | 06/10/11 | 0 | 20/09/12 | 3 |
| 83BT77 | Sillans-la-Cascade | 06/10/11 | 0 | 20/09/12 | 2 |
| 83BT78 | Le Muy | 05/10/11 | 0 | 17/09/12 | 0 |
| 83BT79 | Callas | 06/10/11 | 0 | 19/09/12 | 2 |
| 83PL1 | Roquebrune-sur-Argens | 05/10/11 | 0 | 17/09/12 | 232 |
| 83PL2 | Barjols | 05/10/11 | 0 | 17/09/12 | 0 |
| 83PS6 | Hyères | 04/10/11 | 0 | 18/09/12 | 0 |

The dashes indicate no collection.

**Table S3 Details of the entomological surveys realized in Corsica in 2010 and 2012.**

|  |  | **2010** | | **2012** | |
| --- | --- | --- | --- | --- | --- |
| **Code** | **Locations** | **Collection date** | **Maximum catch per trap per night** | **Collection date** | **Maximum catch per trap per night** |
| Ca1 | Figari | 11/08/2010 | 162240 | 17/07/2012 | 48780 |
| Ca3 | Bastelicaccia | 08/09/2010 | 717 | 13/11/2012 | 58 |
| Cb1 | San-Giuliano | 04/10/2010 | 927 | 15/10/2012 | 1597 |
| Cb3 | Moltifao | 10/08/2010 | 145 | 20/11/2012 | 379 |

**Table S4 Pairwise F_ST_ values between Corsican and Var populations of *C. imicola* for all collected years.**

|  | Ca1_08 | Ca1_10 | Ca1_12 | Ca2_02 | Ca2_08 | Ca3_10 | Ca3_12 | Cb1_02 | Cb1_08 | Cb1_10 | Cb2_02 | Cb2_08 | Cb3_08 | Cb3_10 | Cb3_12 | V1_06 | V1_08 | V1_10 | V1_12 | V2_08 | V2_10 | V2_12 | V3_08 | V3_12 | Sd_12 |
| --- | --- | --- | --- | --- | --- | --- | --- | --- | --- | --- | --- | --- | --- | --- | --- | --- | --- | --- | --- | --- | --- | --- | --- | --- | --- |
| Ca1_02 | -0.0104  (0.970) | -0.0029  (0.572) | -0.0081  (0.946) | 0.0001  (0.329) | 0.0004  (0.538) | 0.0030  (0.453) | 0.0027  (0.360) | -0.0050  (0.682) | 0.0109  (0.016) | -0.0015  (0.078) | 0.0027  (0.043) | -0.0019  (0.660) | -0.0057  (0.627) | -0.0033  (0.541) | 0.0046  (0.199) | **0.0363**  (0.0001) | **0.0306**  (0.0001) | **0.0329**  (0.0001) | **0.0407**  (0.0001) | **0.0212**  (0.0001) | **0.0282**  (0.0001) | 0.0262  (0.0005) | 0.0205  (0.0002) | **0.0334**  (0.0001) | 0.0157  (0.0008) |
| Ca1_08 |  | -0.0075  (0.921) | -0.0090  (0.633) | 0.0025  (0.709) | -0.0036  (0.670) | -0.0029  (0.821) | 0.0002  (0.311) | -0.0091  (0.709) | 0.0054  (0.153) | -0.0042  (0.243) | -0.0081  (0.687) | -0.0045  (0.770) | -0.0084  (0.897) | -0.0104  (0.978) | 0.0024  (0.577) | **0.0408**  (0.0001) | **0.0245**  (0.0001) | **0.0351**  (0.0001) | **0.0382**  (0.0001) | 0.0236  (0.0003) | **0.0289**  (0.0001) | 0.0307  (0.0004) | 0.0246  (0.0007) | **0.0363**  (0.0001) | 0.0097  (0.012) |
| Ca1_10 |  |  | -0.0048  (0.467) | 0.0030  (0.227) | 0.0104  (0.124) | -0.0054  (0.817) | -0.0067  (0.542) | 0.0026  (0.034) | 0.0046  (0.026) | 0.0047  (0.004) | 0.0007  (0.066) | 0.0028  (0.278) | -0.0048  (0.510) | 0.0019  (0.049) | 0.0075  (0.037) | **0.0418**  (0.0001) | **0.0277**  (0.0001) | **0.0335**  (0.0001) | **0.0317**  (0.0001) | **0.0186**  (0.0001) | 0.0181  (0.0001) | **0.0307**  (0.0001) | 0.0227  (0.0004) | **0.0306**  (0.0001) | **0.0215**  (0.0001) |
| Ca1_12 |  |  |  | 0.0046  (0.101) | 0.0038  (0.212) | -0.0021  (0.237) | 0.0062  (0.043) | -0.0020  (0.200) | 0.0123  (0.0004) | 0.0021  (0.098) | -0.0012  (0.023) | 0.0023  (0.286) | 0.0020  (0.037) | -0.0040  (0.289) | 0.0027  (0.060) | **0.0468**  (0.0001) | **0.0362**  (0.0001) | **0.0424**  (0.0001) | **0.0506**  (0.0001) | **0.0332**  (0.0001) | **0.0334**  (0.0001) | **0.0400**  (0.0001) | **0.0352**  (0.0001) | **0.0431**  (0.0001) | 0.0194  (0.0007) |
| Ca2_02 |  |  |  |  | 0.0006  (0.544) | 0.0056  (0.048) | 0.0044  (0.112) | 0.0104  (0.088) | 0.0201  (0.008) | 0.0085  (0.013) | 0.0104  (0.018) | 0.0038  (0.235) | 0.0032  (0.537) | 0.0087  (0.085) | 0.0066  (0.374) | **0.0427**  (0.0001) | **0.0406**  (0.0001) | **0.0347**  (0.0001) | **0.0427**  (0.0001) | **0.0296**  (0.0001) | **0.0356**  (0.0001) | **0.0433**  (0.0001) | **0.0253**  (0.0001) | **0.0361**  (0.0001) | 0.0182  (0.0005) |
| Ca2_08 |  |  |  |  |  | 0.0126  (0.013) | 0.0099  (0.059) | -0.0002  (0.409) | 0.0180  (0.006) | 0.0097  (0.075) | -0.0003  (0.291) | -0.0016  (0.488) | 0.0026  (0.153) | 0.0038  (0.134) | 0.0108  (0.025) | **0.0267**  (0.0001) | **0.0181**  (0.0001) | **0.0229**  (0.0001) | **0.0349**  (0.0001) | **0.0232**  (0.0001) | **0.0297**  (0.0001) | **0.0239**  (0.0001) | **0.0227**  (0.0001) | **0.0290**  (0.0001) | 0.0158  (0.001) |
| Ca3_10 |  |  |  |  |  |  | 0.0045  (0.302) | 0.0118  (0.002) | 0.0148  (0.0004) | 0.0050  (0.001) | -0.0011  (0.170) | 0.0019  (0.195) | -0.0033  (0.409) | 0.0012  (0.121) | -0.0016  (0.729) | **0.0479**  (0.0001) | **0.0399**  (0.0001) | **0.0374**  (0.0001) | **0.0438**  (0.0001) | **0.0303**  (0.0001) | **0.0343**  (0.0001) | **0.0430**  (0.0001) | **0.0329**  (0.0001) | **0.0408**  (0.0001) | 0.0090  (0.003) |
| Ca3_12 |  |  |  |  |  |  |  | 0.0037  (0.069) | **0.0102**  (0.0001) | 0.0067  (0.011) | 0.0069  (0.061) | -0.0007  (0.449) | -0.0003  (0.229) | 0.0039  (0.123) | 0.0104  (0.089) | **0.0425**  (0.0001) | **0.0275**  (0.0001) | **0.0307**  (0.0001) | **0.0265**  (0.0001) | **0.0161**  (0.0001) | **0.0226**  (0.0001) | **0.0337**  (0.0001) | 0.0223  (0.0004) | **0.0345**  (0.0001) | 0.0172  (0.0002) |
| Cb1_02 |  |  |  |  |  |  |  |  | 0.0116  (0.070) | 0.0048  (0.378) | 0.0030  (0.091) | 0.0049  (0.038) | 0.0023  (0.080) | 0.0005  (0.174) | 0.0178  (0.010) | **0.0381**  (0.0001) | **0.0259**  (0.0001) | **0.0438**  (0.0001) | **0.0497**  (0.0001) | **0.0278**  (0.0001) | **0.0312**  (0.0001) | **0.0325**  (0.0001) | **0.0355**  (0.0001) | **0.0421**  (0.0001) | **0.0199**  (0.0001) |
| Cb1_08 |  |  |  |  |  |  |  |  |  | 0.0089  (0.048) | 0.0105  (0.006) | 0.0137  (0.002) | 0.0037  (0.050) | 0.0128  (0.002) | 0.0290  (0.0004) | **0.0577**  (0.0001) | **0.0276**  (0.0001) | **0.0409**  (0.0001) | **0.0316**  (0.0001) | **0.0395**  (0.0001) | **0.0202**  (0.0001) | **0.0378**  (0.0001) | **0.0238**  (0.0001) | **0.0575**  (0.0001) | **0.0289**  (0.0001) |
| Cb1_10 |  |  |  |  |  |  |  |  |  |  | -0.0003  (0.060) | 0.0020  (0.074) | 0.0014  (0.008) | 0.0020  (0.020) | 0.0061  (0.013) | **0.0670**  (0.0001) | **0.0507**  (0.0001) | **0.0577**  (0.0001) | **0.0484**  (0.0001) | **0.0445**  (0.0001) | **0.0512**  (0.0001) | **0.0543**  (0.0001) | **0.0396**  (0.0001) | **0.0603**  (0.0001) | 0.0012  (0.009) |
| Cb2_02 |  |  |  |  |  |  |  |  |  |  |  | -0.0023  (0.240) | 0.0007  (0.044) | -0.0028  (0.169) | 0.0028  (0.128) | **0.0399**  (0.0001) | **0.0241**  (0.0001) | **0.0345**  (0.0001) | **0.0362**  (0.0001) | **0.0261**  (0.0001) | **0.0302**  (0.0001) | **0.0307**  (0.0001) | **0.0254**  (0.0001) | **0.0368**  (0.0001) | 0.0019  (0.001) |
| Cb2_08 |  |  |  |  |  |  |  |  |  |  |  |  | -0.0074  (0.490) | -0.0037  (0.732) | 0.0019  (0.298) | **0.0347**  (0.0001) | **0.0235**  (0.0001) | **0.0277**  (0.0001) | **0.0372**  (0.0001) | 0.0147  (0.0003) | **0.0317**  (0.0001) | **0.0291**  (0.0001) | 0.0204  (0.001) | **0.0319**  (0.0001) | 0.0018  (0.001) |
| Cb3_08 |  |  |  |  |  |  |  |  |  |  |  |  |  | -0.0023  (0.264) | 0.0057  (0.695) | **0.0369**  (0.0001) | **0.0240**  (0.0001) | **0.0243**  (0.0001) | **0.0288**  (0.0001) | 0.0138  (0.0008) | **0.0205**  (0.0001) | 0.0215  (0.001) | 0.0144  (0.002) | **0.0295**  (0.0001) | 0.0071  (0.021) |
| Cb3_10 |  |  |  |  |  |  |  |  |  |  |  |  |  |  | -0.0036  (0.518) | **0.0443**  (0.0001) | **0.0326**  (0.0001) | **0.0351**  (0.0001) | **0.0429**  (0.0001) | **0.0265**  (0.0001) | **0.0372**  (0.0001) | **0.0376**  (0.0001) | **0.0281**  (0.0001) | **0.0415**  (0.0001) | 0.0102  (0.023) |
| Cb3_12 |  |  |  |  |  |  |  |  |  |  |  |  |  |  |  | **0.0512**  (0.0001) | **0.0489**  (0.0001) | **0.0392**  (0.0001) | **0.0501**  (0.0001) | **0.0291**  (0.0001) | **0.0482**  (0.0001) | **0.0457**  (0.0001) | **0.0363**  (0.0001) | **0.0385**  (0.0001) | 0.0117  (0.0025) |
| V1_06 |  |  |  |  |  |  |  |  |  |  |  |  |  |  |  |  | **0.0069**  (0.0001) | **0.0086**  (0.0001) | **0.0261**  (0.0001) | 0.0081  (0.173) | 0.0157  (0.012) | 0.0069  (0.426) | 0.0168  (0.004) | -0.0005  (0.384) | **0.0635**  (0.0001) |
| V1_08 |  |  |  |  |  |  |  |  |  |  |  |  |  |  |  |  |  | 0.0016  (0.296) | 0.0083  (0.018) | 0.0034  (0.592) | 0.0034  (0.113) | 0.0058  (0.170) | 0.0089  (0.028) | 0.0181  (0.004) | **0.0516**  (0.0001) |
| V1_10 |  |  |  |  |  |  |  |  |  |  |  |  |  |  |  |  |  |  | 0.0018  (0.149) | 0.0000  (0.461) | 0.0041  (0.099) | 0.0015  (0.159) | -0.0003  (0.265) | 0.0092  (0.011) | **0.0548**  (0.0001) |
| V1_12 |  |  |  |  |  |  |  |  |  |  |  |  |  |  |  |  |  |  |  | 0.0130  (0.0028) | 0.0060  (0.060) | 0.0139  (0.002) | 0.0008  (0.054) | 0.0206  (0.002) | **0.0559**  (0.0001) |
| V2_08 |  |  |  |  |  |  |  |  |  |  |  |  |  |  |  |  |  |  |  |  | 0.0061  (0.171) | -0.0017  (0.687) | 0.0047  (0.119) | -0.0011  (0.259) | **0.0418**  (0.0001) |
| V2_10 |  |  |  |  |  |  |  |  |  |  |  |  |  |  |  |  |  |  |  |  |  | -0.0029  (0.933) | -0.0028  (0.718) | 0.0115  (0.021) | **0.0617**  (0.0001) |
| V2_12 |  |  |  |  |  |  |  |  |  |  |  |  |  |  |  |  |  |  |  |  |  |  | -0.0012  (0.607) | -0.0004  (0.030) | **0.0584**  (0.0001) |
| V3_08 |  |  |  |  |  |  |  |  |  |  |  |  |  |  |  |  |  |  |  |  |  |  |  | 0.0119  (0.026) | **0.0472**  (0.0001) |
| V3_12 |  |  |  |  |  |  |  |  |  |  |  |  |  |  |  |  |  |  |  |  |  |  |  |  | **0.0604**  (0.0001) |

Significant F_ST_ are represented in bold. P-value adjusted at the nominal level (5%) after Bonferroni correction is 0.0001. Details on sample codes are given in Table 1.
